# Severe autoinflammatory disease caused by mutation in a gene controlling actin cytoskeletal dynamics and cure with allogeneic haematopoetic stem cell transplantation

**DOI:** 10.1186/1546-0096-13-S1-O19

**Published:** 2015-09-28

**Authors:** A Standing, D Malinova, J Record, D Moulding, M Blundell, K Nowak, H Jones, E Omoyinmi, S Nanthapisal, S Melo Gomes, Y Hong, N Klein, D Eleftheriou, A Thrasher, P Brogan

**Affiliations:** 1 UCL Institute of Child Health, IIIP, London, UK

## Introduction

The actin cytoskeleton is crucial at many junctures of normal immune function, and consequently there are many immune specific regulators of actin dynamics. A growing number of primary immunodeficiencies are being defined as caused by mutations in the genes encoding these regulators. In addition to immunodeficiency, immune dysregulation and autoinflammation are increasingly recognised to arise from defects within this pathway.

## Objectives

To use next generation sequencing, and functional studies to identify the genetic cause for a severe unclassified autoinflammatory disease mimicking Behcet's disease in a consanguineous family.

## Patients and methods

Two affected children in a consanguineous Pakistani kindred suffered from an unclassified autoinflammatory syndrome presenting in the first year of life with: severe oral ulceration resulting in scarring; perianal cutaneous ulceration; severe sterile recurrent fevers; and intermittent episodes of thrombocytopenia associated with intercurrent (presumed) viral infections. Both children were partially responsive to corticosteroids; DMARDS were largely ineffective. TNF-alpha blockade was ineffective; IL-1-beta blockade with Anakinra was partially effective. Despite that the older sibling died at the age of 14.5 years from severe sterile systemic inflammation, thrombocytopaenia, and multi-organ failure. In view of this, the younger (index) sibling underwent allogeneic haematopoietic stem cell transplantation (HSCT) at the age of seven years, and is 100% engrafted 23 months later with complete cure of her illness.

DNA from the two patients, two unaffected siblings and their parents were genotyped for homozygosity mapping. One of the affected patients was exome sequenced, and the homozygous regions scrutinised. Variants of interest segregating with disease were confirmed with Sanger sequencing. Patient cells were analysed by flow cytometry and confocal microscopy to visualise polymerised actin levels and phagocytic activity of dendritic cells (DCs). Cellular motility was assessed using a Dunn chamber. These assays were also used to assess THP1 monocytic cell lines modified with shRNA and derived to macrophage-like and DC-like phenotypes. Wild-type and mutant sequence were tagged with mCherry fluorescent protein and overexpressed in HEK393T cells.

## Results

The index case demonstrated normal T-cell activation to PHA but absent T-cell response to anti-CD3, highly suggestive of a defect in actin-regulated organisation of the immune synapse. A homozygous missense mutation was identified in a gene encoding a regulator of actin stability. CD20+ and CD56+ (but not CD3+) cells showed increased levels of polymerised actin. Dendritic cell and neutrophil motility was disrupted. The mutation identified resulted caused abnormal cellular localisation of the encoded actin-regulating protein.

## Conclusion

We describe a novel monogenic autoinflammatory disease with sterile inflammation and intermittent thrombocytopenia in association with periodic fever. This resulted from a mutation in a regulator of the actin cytoskeleton, and was resistant to conventional immunosuppression, though ultimately cured by HSCT.

